# Systematic Review and Meta-Analysis of the Clinical Features Associated With Seronegative Autoimmune Encephalitis

**DOI:** 10.1212/NXI.0000000000200540

**Published:** 2026-01-07

**Authors:** Leonardo Di Cosmo, Smaila Mulic-Al Bunni, Yihui Goh, Justyna Przybysz, Victor C. Mgbachi, Hannah Fox, Jonathan Cleaver, Patrick J. Waters, Hana Boček, Thashi Chang, Nilanka Wickramasinghe, Alessandra Morano, Sarosh R. Irani, Soon-Tae Lee, Sophie N. M. Binks, Adam E. Handel

**Affiliations:** 1 Humanitas University, School of Medicine, Pieve Emanuele, Milan, Italy;; 2 Oxford Autoimmune Neurology Group, Nuffield Department of Clinical Neurosciences, University of Oxford, United Kingdom;; 3 Department of Neurology, Seoul National University Hospital, Seoul National University College of Medicine, South Korea;; 4 Division of Neurology, Department of Medicine, National University Health System, Singapore;; 5 Department of Medicine, Yong Loo Lin School of Medicine, National University of Singapore, Singapore;; 6 Department of Neurology, Oxford University Hospitals NHS Foundation Trust, United Kingdom;; 7 Oxford Autoimmune Neurology Diagnostic Laboratory, Nuffield Department of Clinical Neurosciences, University of Oxford, United Kingdom;; 8 Department of Neurology, Second Faculty of Medicine, Charles University, Prague, Czech Republic;; 9 Motol University Hospital, Prague, Czech Republic;; 10 Department of Clinical Medicine, Faculty of Medicine, University of Colombo, Sri Lanka;; 11 Department of Physiology, Faculty of Medicine, University of Colombo, Sri Lanka;; 12 Epilepsy Unit, Department of Human Neurosciences, “Sapienza” University of Rome, Italy; and; 13 Departments of Neurology and Neurosciences, Mayo Clinic, Jacksonville, FL.

## Abstract

**Background and Objectives:**

Seronegative autoimmune encephalitis (AE), defined by an appropriate clinical phenotype in the absence of known neuronal autoantibodies, poses diagnostic and therapeutic challenges due to clinical heterogeneity and lack of definitive biomarkers. We conducted a systematic review and meta-analysis of individual patient data to characterize the phenotypes, treatment responses, and prognostic factors in seronegative AE.

**Methods:**

We included 213 cases from 30 studies published between 2014 and 2024 and 11 from a local cohort meeting Graus criteria for seronegative AE. We extracted details on clinical and paraclinical features, immunotherapy, and outcomes measured via the modified Rankin Scale (mRS) and Clinical Assessment Scale for Autoimmune Encephalitis (CASE). Multivariate regression and dimensionality reduction analyses identified prognostic markers.

**Results:**

Of 224 patients (median age 49 years, 50.9% male), 72 (32.1%) had limbic encephalitis (LE) and 152 (67.9%) had antibody-negative probable AE (ANPRA). Good outcome (mRS score ≤2) was more common in LE (49/72, 68.1%) than in ANPRA (76/154, 50.0%) (*p* < 0.05). Delayed immunotherapy was associated with an increased risk of poor outcome. Additional predictors of poor prognosis included age older than 60 years, the ANPRA subtype, an underlying tumor, striatocapsular or thalamic involvement on MRI, and presentation with refractory status epilepticus. Dimensionality and clustering analysis identified heterogeneity among seronegative AE, with 3 distinct subtypes.

**Discussion:**

Seronegative AE comprises clinically and prognostically distinct subtypes. Early immunotherapy is the key modifiable factor influencing outcome. We advocate for biomarker discovery and prospective, systematically reported cohort studies to improve stratification and treatment strategies in this diagnostically challenging population.

## Introduction

Autoimmune encephalitis (AE) is a group of rapidly progressive, immune-mediated inflammatory disorders frequently associated with autoantibodies targeting CNS intracellular and surface antigens.^1,2^ AE presents with diverse clinical manifestations, including altered mental status, memory impairment, psychiatric symptoms, seizures, and other focal neurologic deficits. Detection of autoantibodies is an integral part of AE diagnostic criteria,^3,4^ which can inform the likely response to and duration of immunotherapy.^5^

However, despite research efforts, around a third to a half of patients with AE do not have detectable antibodies against known neuronal antigens.^6^ Therefore, diagnosis relies substantially on clinical and paraclinical findings.^3^ Clinical and paraclinical diagnostic criteria for seronegative AE are likely specific but have relatively low sensitivity.^7^ Identifying additional biomarkers is urgent to ensure that patients with AE are not missed. For example, only around a quarter of patients with NMDAR-antibody encephalitis would meet diagnostic criteria for seronegative AE without a positive antibody result.^8^ The absence of a definitive biomarker in seronegative AE results in clinical heterogeneity and diagnostic uncertainty, complicating therapeutic decision making and prognostication.^9,10^

To help address these challenges, this systematic review and meta-analysis synthesizes individual patient data from the available literature (n = 213) and integrates these with a local, previously unreported cohort of patients (n = 11) with seronegative AE. Through this approach, we aim to delineate further the clinical and paraclinical aspects of seronegative AE subtypes, assess responsiveness to immunotherapy, and identify prognostic markers in a large cohort.

## Methods

### Literature Review Design

This systematic review was registered with PROSPERO (CRD42024555388) and conducted following Preferred Reporting Items for Systematic Reviews and Meta-Analyses guidelines.

### Search Strategy

A comprehensive search of PubMed, EMBASE, and Cochrane databases was performed for studies published between January 1, 2014, and June 1, 2024. The search used the following terms in titles, abstracts, and keywords: (“autoantibody negative” OR “autoantibody-negative” OR “seronegative” OR “sero-negative” OR “antibody negative” OR “antibody-negative”) AND (“limbic” OR “encephalitis”).

### Eligibility Criteria

Two reviewers (S.M.B. and L.D.C.) independently screened titles and abstracts, with disagreements resolved by a third reviewer (A.E.H.). Reference lists of included studies were manually searched for additional studies meeting the inclusion criteria.

Inclusion criteria were as follows: (1) case reports, case series, and cohort or quasi-experimental studies of any size reporting seronegative autoimmune encephalitis (AE); (2) adult patients (≥18 years) or studies stratified by age; (3) diagnosis of probable antibody-negative limbic encephalitis (LE) or antibody-negative probable AE (ANPRA) as per Graus criteria^3^; (4) reporting of clinical outcomes (physical or neurologic function) before and after treatment.

Exclusion criteria included the following: (1) studies focused solely on pediatric populations or without age stratification; (2) inclusion of patients not meeting Graus criteria^3^; (3) insufficient reporting of outcomes after immunotherapy, or lacking sufficient clinical detail to derive these outcomes; (4) non–peer-reviewed literature, including reviews, editorials, and abstracts; (5) non-English publications; (6) studies lacking individual patient-level data.

### Data Extraction

Patient-level data were extracted into a standardized spreadsheet capturing study year, design, demographics, clinical presentation (clinical features, CSF and blood investigations, infectious screening, autoimmune panels, and MRI/EEG characteristics), treatment, and outcomes at follow-up (eTable 1). Relapses were defined as cases newly meeting Graus criteria for possible autoimmune encephalitis at least 3 months after onset. All data were independently extracted by 2 authors (S.M.B. and L.D.C.). Incomplete cases prompted contact with the study authors. Discrepancies were resolved through discussion or senior adjudication (S.N.M.B. and A.E.H.).

### Quality Assessment

Two reviewers (S.M.B. and L.D.C.) independently assessed study quality using the Joanna Briggs Institute Critical Appraisal Checklist for case-based studies and the Newcastle-Ottawa Scale (NOS) for cohort studies. Disagreements were discussed or resolved with senior authors if necessary.

### Outcome Measures

The primary outcome was a change in functional status, which was assessed via the modified Rankin Scale (mRS) and Clinical Assessment Scale for Autoimmune Encephalitis (CASE). mRS scores were dichotomized into good (≤2) and poor (≥3) outcomes. Secondary outcomes included examining known prognostic factors, including those identified by the RAPID score^11^; characterizing clinical/paraclinical features of seronegative AE subtypes; and identifying associations with seizure recurrence, relapse, or return to work. S.M.B. and L.D.C. reviewed cases with incomplete data to determine whether estimation of outcome measures was feasible; otherwise, these were excluded.

### Local Cohort Study: Participants and Antibody Determination

We supplemented published cases with patients meeting Graus criteria for probable seronegative AE from the John Radcliffe Hospital, Oxford (December 2015–July 2024).

To detect novel autoantibodies, live cultured hippocampal neurons at day in vitro 25 and fixed adult rat brain sections were incubated with serum/CSF samples and visualized, as previously described.^12^

### Standard Protocol Approvals, Registrations, and Patient Consents

This study was approved by the Yorkshire and Humber Research Ethics Committee (REC16/YH/0013). All participants provided written consent.

### Data Analysis

Statistical analysis was performed using R v4.4.1. Univariate analysis assessed associations between clinical variables and outcomes: chi-square or Fisher exact tests for categorical data, using the Fisher exact test when expected cell counts were ≤5 in any group, and the Mann-Whitney *U* test for continuous data. Benjamini-Hochberg correction was applied for multiple testing.

Generalized linear models evaluated associations between clinical features and outcomes using logistic and linear regression. Missing data were addressed using multivariate imputation by chained equations, with a 25% missingness threshold per variable. Variables with a variance inflation factor > 2.5 were excluded for multicollinearity. Stepwise regression (bidirectional) refined model selection. Model strength was tested via bootstrapping, area under the curve (AUC), and pseudo-R^2^. Statistical significance was set at *p* < 0.05 and was reported as corrected *p* values throughout, where appropriate.

### Data Availability

Anonymized data from this study will be made available to any qualified investigator on reasonable request.

## Results

### Data Extraction

**Figure 1:**
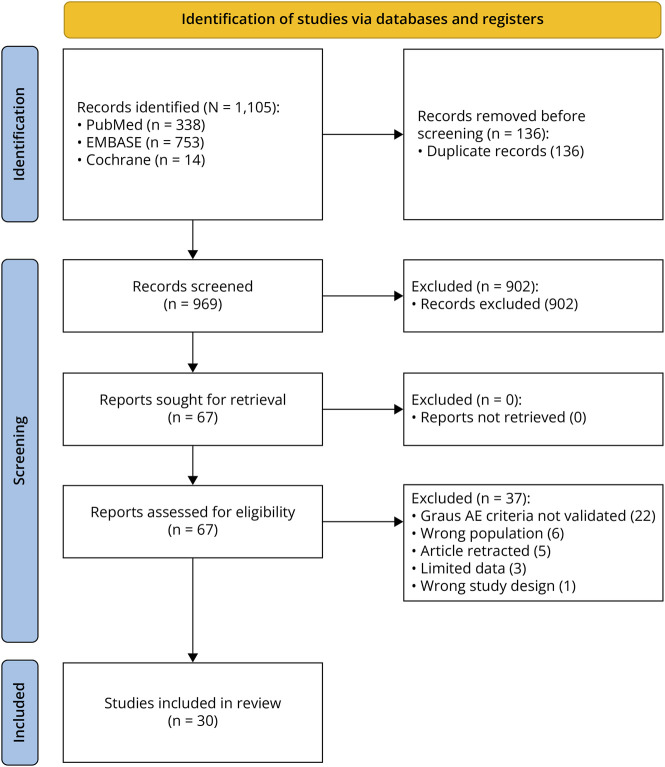
PRISMA Flowchart Describing Article Screening, Selection, and Extraction Process A total of 224 cases were included, comprising 213 cases from the literature (shown below) and 11 from the local Oxford cohort. PRISMA = Preferred Reporting Items for Systematic Reviews and Meta-Analyses.

Our initial search identified 1,105 records across 3 databases. After removing 136 duplicate records, we screened the abstracts of the remaining 969 studies, excluding 902 studies that did not meet the inclusion criteria. The full-text screening of the remaining 67 articles resulted in the exclusion of 37 studies for the following reasons: exclusively pediatric population or inability to differentiate pediatric and nonpediatric populations in results (n = 6), article was retracted (n = 5), insufficient clinically relevant data (n = 3), and study design not aligned with inclusion criteria (n = 1); the remaining studies did not meet the Graus criteria for seronegative AE diagnosis (n = 22). After full-text screening, 30 studies met the inclusion criteria, comprising 21 case reports, 3 case series, and 6 cohort studies (Figure 1 and eTable 2).

The summary of the risk-of-bias assessments for included studies are provided in the supplementary material (eFigure 1).

### Clinical and Paraclinical Phenotypes of Antibody-Negative Encephalitis

Initially, 280 patients with suspected seronegative AE were identified: 260 from the literature and 20 from our cohort. After review, 9 local and 47 literature patients were excluded because of insufficient data or clinical presentations not fulfilling the Graus criteria for probable seronegative AE. A detailed breakdown of exclusions, including patients reclassified under alternative AE etiologies, is given in the supplementary materials (eFigure 2). For the remaining patients, the diagnostic combinations used to classify ANPRA and LE are summarized in eTable 3 and the rates of mRS and CASE reporting are presented in eTable 4.

The final dataset included 224 confirmed patients with seronegative AE, with a median age across the cohort of 49 years (range 18–86), and 114 of 224 were men (50.9%) (Table 1). At first admission, the median mRS score was 4 (range 1–5/6) and the median CASE score was 11 (range 2–27/27). Based on clinical profiles, 72 (32.1%) were classified as LE and 152 (67.9%) as ANPRA (Table 1).

**Table 1:** Comparison of Clinical Profiles at Onset Between Patients With LE and ANPRA

Variable	Total (n = 224)	ANPRA (n = 152)	LE (n = 72)	*p* Value
Clinical profiles				
Male sex	114 (50.9%)	80 (52.6%)	34 (47.2%)	0.70
Patient age	49 (18–86)	47 (18–86)	53.5 (20–82)	0.22
Age older than 60 years	71 (31.7%)	46 (30.3%)	25 (34.7%)	0.76
Tumor	18 (8%)	6 (3.9%)	12 (16.7%)	<0.01
Baseline mRS score value	4 (1–5)	5 (2–5)	4 (1–5)	<0.001
Baseline CASE score value	11 (2–27)	12 (2–27)	7.5 (2–25)	<0.001
Symptom profiles				
Seizure	162 (72.3%)	115 (75.7%)	47 (65.3%)	0.27
Memory dysfunction	200 (89.3%)	135 (88.8%)	65 (90.3%)	1.00
Psychiatric symptoms	146 (65.2%)	104 (68.4%)	42 (58.3%)	0.31
Mood disorder–related symptoms	31 (13.8%)	10 (6.6%)	21 (29.2%)	<0.001
Psychosis	22 (9.8%)	10 (6.6%)	12 (16.7%)	0.07
Impaired consciousness	151 (67.4%)	119 (78.3%)	32 (44.4%)	<0.001
Language problems	124 (55.4%)	104 (68.4%)	20 (27.8%)	<0.001
Movement disorders	59 (26.3%)	50 (32.9%)	9 (12.5%)	<0.01
Weakness	84 (37.5%)	70 (46.1%)	14 (19.4%)	<0.001
Brainstem dysfunction	77 (34.4%)	67 (44.1%)	10 (13.9%)	<0.001
Gait instability and ataxia	117 (52.2%)	99 (65.1%)	18 (25%)	<0.001

Abbreviations: ANPRA = antibody-negative probable AE; CASE = Clinical Assessment Scale for Autoimmune Encephalitis; LE = limbic encephalitis; mRS = modified Rankin Scale.

Variables included in the original analysis have been reduced to focus on the most significant characteristics. *p* Values were adjusted using Benjamini-Hochberg correction for multiple comparisons. Data are presented as count (percentage) for categorical variables or median (range) for numerical variables.

Common symptoms at onset included memory dysfunction (200/224, 89.3%), seizures (162/224, 72.3%), impaired consciousness (151/224, 67.4%), and psychiatric symptoms (146/224, 65.2%), which comprised paranoia, hallucinations, agitation, and affective lability (Table 1). The phenotypic differences distinguishing LE and ANPRA were further explored. Mood disorder symptoms, characterized by presentations of depression and anxiety, were more frequent in LE (21/72, 29.2%) vs ANPRA (10/152, 6.6%; *p* < 0.001). Patients with ANPRA more often presented with gait ataxia (99/152, 65.1% vs 18/72, 25.0%; *p* < 0.001), language problems (104/152, 68.4% vs 20/72, 27.8%; *p* < 0.001), brainstem dysfunction (67/152, 44.1% vs 10/72, 13.9%; *p* < 0.001), weakness (70/152, 46.1% vs 14/72, 19.4%; *p* < 0.001), and movement disorders (50/152, 32.9% vs 9/72, 12.5%; *p* < 0.01), including parkinsonism, dyskinesia, and dystonia. Other symptoms showed no statistically significant differences (Table 1).

In accordance with diagnostic criteria, medial temporal lobe involvement was identified in all patients with LE (72/72, 100%) compared with only half of the ANPRA group (unilateral only: 80/152, 52.6%; *p* < 0.001) (Table 2). Additional distinct imaging features emerged between the subtypes, with infratentorial MRI abnormalities more common in ANPRA (20/152, 13.2%) than in LE (1/72, 1.4%; *p* < 0.05). Representative MRI findings from the local seronegative cohort are displayed in eFigure 3.

**Table 2:** Comparison of Paraclinical Profiles at Onset Between Patients With LE and ANPRA

Variable	Total (n = 224)	ANPRA (n = 152)	LE (n = 72)	*p* Value
CSF abnormality	206 (92.0%)	152 (100.0%)	54 (75.0%)	< 0.001
CSF protein level g/L	0.6 (0.1–17.0)	0.6 (0.1–4.0)	0.5 (0.2–17.0)	0.16
CSF leukocyte cells/μL	12 (0–2,760)	13 (0–2,760)	10 (0–84)	0.16
CSF oligoclonal band positivity	27 (16.7%)	19 (15.2%)	8 (21.6%)	0.68
Any MRI abnormality	224 (100.0%)	152 (100.0%)	72 (100.0%)	1.00
Medial temporal lobe involvement	152 (67.9%)	80 (52.6%)	72 (100.0%)	< 0.001
Infratentorial involvement	21 (9.4%)	20 (13.2%)	1 (1.4%)	< 0.05
Spinal involvement	6 (2.7%)	6 (3.9%)	0 (0.0%)	0.31
Contrast enhancement	16 (7.1%)	13 (8.6%)	3 (4.2%)	0.53
Any abnormal EEG	131 (84.5%)	78 (83.9%)	53 (85.5%)	1.00
EEG focal seizure	20 (12.9%)	8 (8.6%)	12 (19.4%)	0.17
Refractory status epilepticus	48 (21.4%)	35 (23.0%)	13 (18.1%)	0.68

Abbreviations: ANPRA = antibody-negative probable AE; LE = limbic encephalitis.

Variables included in the original analysis have been reduced to focus on the most significant characteristics. *p* Values were adjusted using Benjamini-Hochberg correction for multiple comparisons. Data are presented as count (percentage) for categorical variables or median (range) for numerical variables.

CSF and EEG profiles were similar across subtypes (Table 2). Oligoclonal bands (OCBs) were tested in 162 patients (37 with LE, 125 with ANPRA), with no significant difference in CSF-restricted OCB positivity (LE: 6/37, 16.2%, vs ANPRA: 18/125, 14.4%; *p* = 0.79) and only 3 patients showing paired positivity in both CSF and serum (eTables 5 and 6). Other CSF parameters, including leucocyte count and protein level, and EEG markers, including total abnormalities, focal seizures, and refractory status epilepticus, showed no significant differences (Table 2).

Treatment patterns varied; corticosteroid use was similar (LE: 60/72, 83.3% vs ANPRA: 121/152, 79.6%; *p* = 0.71), but patients with ANPRA more frequently received IVIG (124/152, 81.6% vs 42/72, 58.3%; *p* < 0.01), rituximab (88/152, 57.9% vs 27/72, 37.5%; *p* < 0.05), and tocilizumab (49/152, 32.2% vs 8/72, 11.1%; *p* < 0.01) (eTable 7). First-line and second-line immunotherapy timings did not differ significantly (eTable 7).

Regarding outcomes, good functional outcomes based on mRS after immunotherapy were less frequent in ANPRA (76/152, 50.0%) than in LE (49/72, 68.1%; *p* < 0.05) (eTable 7). Seizure persistence at follow-up was more common in ANPRA (76/152, 50.0%) than in LE (21/72, 29.2%; *p* < 0.05) (eTable 7). Mortality rates were not significantly different (LE: 10/72, 13.9% vs ANPRA: 17/152, 11.2%; *p* = 0.84) (eTable 7). Data on relapses were available for 154 patients after a median follow-up of 24 months. Relapse rates did not differ between subtypes (LE: 1/38, 2.6% vs ANPRA: 10/116, 8.6%; *p* = 0.45) (eTable 7).

In the local cohort, 3 of 11 samples (27.3%) bound to brain IHC and 1/11 (9.1%) to live neurons (eFigure 4). All patient characteristics, the specific distribution of CSF antibody panels tested across the cohort, identified paraneoplastic conditions, brain IHC results, and neuronal assay results in the local cohort are provided in supplementary materials (eTables 8–12).

### Validation of the RAPID Score

**Figure 2:**
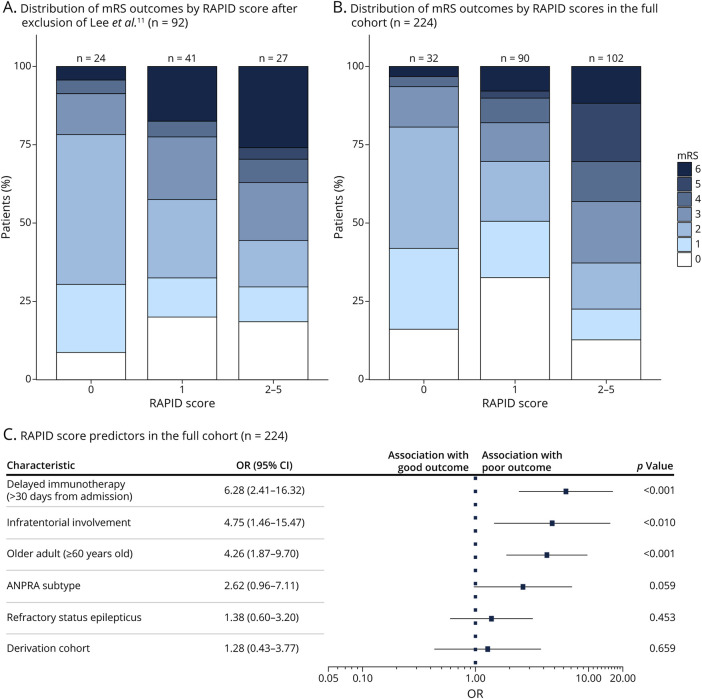
RAPID Scores and Clinical Outcomes Stacked bar plots of mRS outcome scores stratified by RAPID scores across (A) the whole cohort (n = 224) and (B) patients not included in the RAPID score derivation study (n = 92). (C) A forest plot showing mRS scores dichotomized into good (mRS ≤2) or poor (mRS ≥3) outcomes by components of the RAPID score. We included a factor indicating whether each case had been included in the RAPID score derivation cohort. CI = confidence interval; mRS = modified Rankin Scale; OR = odds ratio.

The RAPID score is a previously developed prognostic tool shown to predict 2-year outcomes in patients with seronegative AE based on 5 clinical features: refractory status epilepticus, age at onset of 60 years or older, ANPRA subtype, infratentorial involvement, and delayed immunotherapy (>1 month from hospital admission).^11^ First, we explored the RAPID score for predicting outcome, as measured by final mRS scores after immunotherapy, after excluding those patients originally used to derive the model. This yielded a modest association (RAPID score vs poor final mRS score: Spearman rho = 0.245, *p* = 0.020; Figure 2A and eFigure 5). As expected, the magnitude of this was increased when assessed across the entire cohort, including the original RAPID cohort (Figure 2B; RAPID score vs poor final mRS score: Spearman rho = 0.38, *p* < 0.001). Logistic regression analysis across the whole cohort demonstrated effects on outcome in the same direction as seen in the original RAPID score study (Figure 2C), with a moderate-to-good discriminatory performance (pseudo-R^2^ = 0.205; AUC = 0.79; eFigure 6).

When evaluating the individual variables comprising the RAPID score across the whole cohort, older age at onset (≥60 years), delayed initiation of immunotherapy (>1 month from hospital admission), and infratentorial involvement were each significantly associated with poor functional outcome. By contrast, ANPRA showed a trend toward significance but did not reach the conventional significance threshold while the presence of refractory status epilepticus did not reach statistical significance. To assess for potential bias from including the original RAPID derivation cohort, we included a study-origin variable, which was not independently associated with outcomes (Figure 2C). Univariate analyses for each independent predictor across the derivation and validation cohorts are presented in the supplementary material (eTable 13).

Next, we wanted to identify any additional clinical or paraclinical features driving phenotypic or prognostic heterogeneity in seronegative AE.

### Determinants of Clinical and Prognostic Heterogeneity

**Figure 3:**
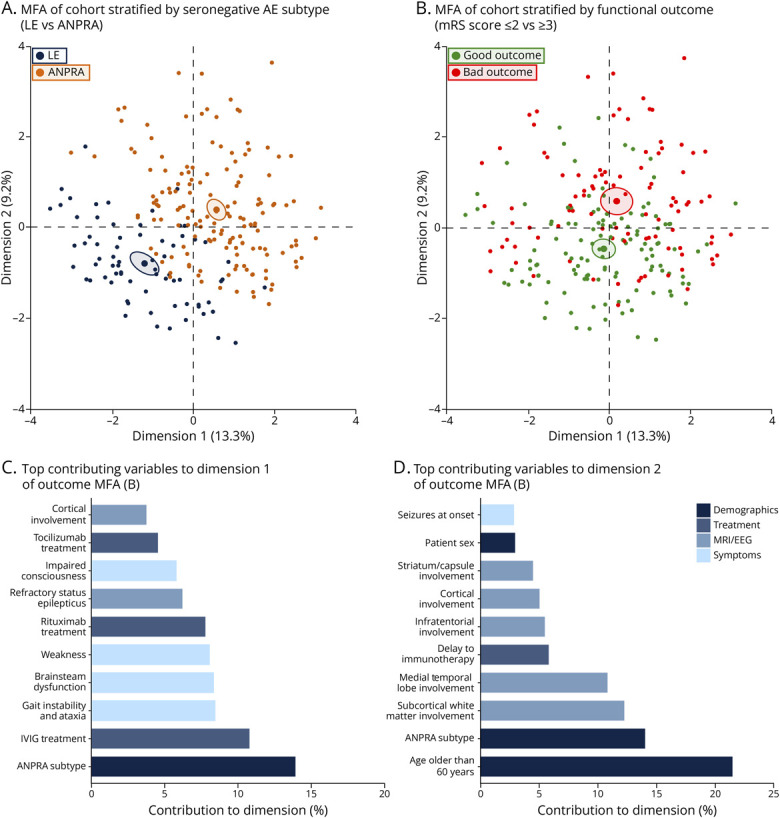
Multiple Factor Analysis of Clinical and Paraclinical Features (Top) MFA stratified by (A) subtypes of seronegative AE and (B) final postimmunotherapy mRS scores. (Bottom) Bar plots showing the top 10 variables contributing to (C) dimension 1 and (D) dimension 2 of the MFA based on final postimmunotherapy mRS scores. Ellipses represent 95% confidence intervals for each group, and the central dots illustrate the centroid of each cluster. mRS = modified Rankin Scale; IVIG = IV immunoglobulin.

Multiple factor analysis (MFA) showed apparent heterogeneity among cases with seronegative AE and eventual clinical outcomes within this cohort (Figure 3, A and B). Factors driving poor prognoses included age older than 60 years, subcortical/white matter involvement, ANPRA subtype, infratentorial involvement, and delayed immunotherapy (Figure 3B; eFigure 7). In addition, cyclophosphamide emerged as a top contributing factor, likely reflecting a treatment profile of more refractory cases. Some but not all of these variables were highlighted in the RAPID score,^11^ suggesting a complex interaction of many factors contributing to the eventual outcome in this cohort. A heatmap applying hierarchical clustering of clinical and paraclinical findings across the cohort revealed distinct clusters corresponding to the LE and ANPRA subtypes (eFigure 8), further supporting the separation observed in the MFA (Figure 3A). Notably, within the ANPRA cluster, 3 phenotypic subgroups were apparent: (1) brainstem/deep brain subgroup with movement disorders and refractory status epilepticus and generally poorer outcomes, (2) subcortical/white matter–predominant group with more variable prognosis, and (3) a heterogeneous seizure-predominant subgroup with relatively favorable outcomes.

**Figure 4:**
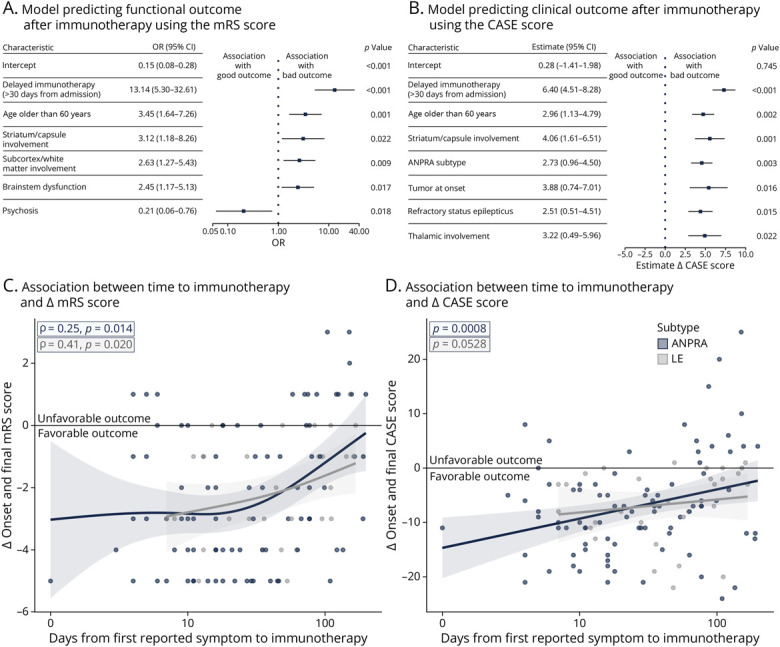
Factors Associated With Clinical Outcomes (Top) Regression models identifying predictors of poor clinical outcomes derived through stepwise regression of relevant clinical and paraclinical variables across the cohort. Forest plots show (A) odds ratios (ORs) and 95% CI for variables associated with a poor mRS outcome and (B) estimates and 95% CI associated with the final CASE score. (Bottom) Scatter plots of the association between time to immunotherapy (days between the first reported symptom and initiation of immunotherapy) and change in functional outcome from onset to final postimmunotherapy follow-up as measured by mRS (C) or CASE (D) scores. The data are stratified by LE and ANPRA, and negative deflections reflect better outcomes. ANPRA = antibody-negative probable AE; CASE = Clinical Assessment Scale for Autoimmune Encephalitis; CI = confidence interval; LE = limbic encephalitis; mRS = modified Rankin Scale; OR = odds ratio.

Therefore, we used stepwise regression modeling to better understand the factors independently influencing prognosis in antibody-negative encephalitis. Age older than 60 years, delayed immunotherapy, brainstem dysfunction, and striatocapsular or subcortical/white matter involvement were significant predictors of poor mRS scores, whereas psychosis at onset was associated with a better outcome (pseudo-R^2^ = 0.298; AUC = 0.842; Figure 4A; eFigure 9).

However, the mRS score captures only a limited proportion of AE-associated disability.^13-15^ Therefore, we used the CASE score as a more granular outcome measure. Linear regression modeling identified age older than 60 years, time to immunotherapy, the ANPRA subtype, an underlying tumor, striatocapsular or thalamic involvement on MRI, or presentation with refractory status epilepticus as factors associated with worse CASE scores (pseudo-R^2^ = 0.349; Figure 4B). We also compared patients with and without relapses and found that CSF protein (relapsing: median 0.9 [range 0.4–3.4], nonrelapsing: 0.6 [0.1–4.0], *p* < 0.05) and leukocyte counts (relapsing: 31 [8–670], nonrelapsing 10 [0–2,760], *p* < 0.05) were elevated in relapsing cases (eTable 14).

The common modifiable factor in both analyses was a delay in immunotherapy. Univariate analysis showed a consistent direction of effect between the time from symptom onset to immunotherapy and outcomes in both ANPRA and LE subgroups (Figure 4, C and D). This correlation was consistent across mRS (ANPRA: ρ = 0.25, *p* = 0.014, vs LE: ρ = 0.41, *p* = 0.020) and CASE (*p* < 0.001 vs *p* = 0.0528) (Figure 4, C and D). In addition, we examined the effect of a delayed immunotherapy on other outcomes, noting a trend toward increased seizure persistence at follow-up, although this did not reach significance (*p* = 0.063; eFigure 10).

**Figure 5:**
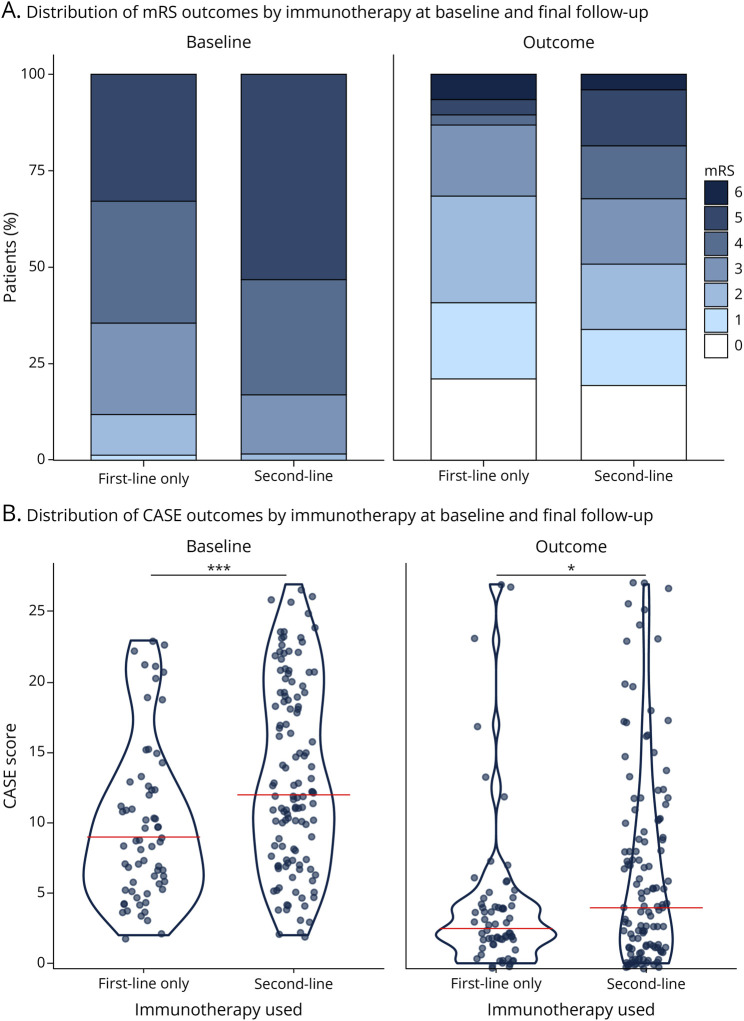
Response to Immunotherapy (Top) Stacked bar plots showing the distribution of mRS scores at baseline (A) and at final follow-up (B), stratified by patients receiving only first-line immunotherapy vs those receiving both first-line and second-line treatments. (Bottom) Violin plots depicting CASE scores at baseline (A) and final follow-up (B), stratified by immunotherapy regimen. Statistical significance is indicated above each comparison (*p < 0.05, ***p < 0.001). CASE = Clinical Assessment Scale for Autoimmune Encephalitis; mRS = modified Rankin Scale.

Given the contribution of immunotherapy to outcomes in this cohort, we compared patients receiving only first-line treatments with those escalated to second-line agents. Patients receiving second-line immunotherapy presented with greater disability at first presentation (median baseline mRS score 5 [IQR 4–5] vs 4 [IQR 3–5], *p* < 0.001; median baseline CASE score 12 [IQR 7.25–19] vs 9 [IQR 5.75–12.25], *p* < 0.001) (Figure 5, A and C). Despite their greater initial disability, both cohorts achieved similar eventual outcomes at final follow-up, with those requiring first-line therapies alone achieving the same median outcome, albeit with significantly better mRS and CASE scores at the group level: the median mRS score was 2 (IQR 1–4) in the second-line group vs 2 (IQR 1–3) in the first-line group (*p* = 0.049), and the median CASE score was 4 (IQR 1–9.75) in the second-line group vs 2.5 (IQR 1.75–4) in the first-line group (*p* = 0.043) (Figure 5, B and D). This indicates the importance of second-line immunotherapy in more severe cases of seronegative AE, allowing patients to attain outcomes similar to those of individuals with less severe initial presentations.

## Discussion

Our individual patient data meta-analysis curated a large cohort of seronegative AE cases. We validated some core elements of the RAPID score while identifying additional prognostic factors, described phenotypic heterogeneity within ANPRA, emphasized the importance of timely immunotherapy, and underscored strict application of Graus criteria to avoid both underdiagnosis and overdiagnosis.

We assessed the generalizability of the RAPID model derived by Lee et al.^11^ by applying it to a broader cohort. The overall RAPID score was associated with measures of outcome in our validation cohort (Spearman rho = 0.245, *p* = 0.02). Three variables within the RAPID score were confirmed as significant predictors of poor outcome: delay in immunotherapy (>30 days from admission), infratentorial involvement, and age older than 60 years. The ANPRA subtype showed a trend toward worse outcomes; however, this did not reach significance. Stepwise analyses in the expanded cohort identified age older than 60 years and delayed immunotherapy as consistent predictors of both poorer mRS and CASE outcomes. Additional markers linked to worse outcomes included brainstem dysfunction, subcortical/white matter involvement, underlying malignancy, and thalamic involvement.

Hierarchical clustering of clinical and paraclinical features confirmed that seronegative AE shows clear segregation between the 2 main subtypes. As expected, LE is characterized by medial temporal lobe involvement, memory dysfunction, and seizures. Patients with ANPRA display greater heterogeneity, but we identified 3 distinct subgroups: one characterized by significant brainstem and deep brain structure involvement, often with associated movement disorders and refractory status epilepticus, and linked to poorer outcomes, another heterogeneous group with prominent seizures, typically with more favorable outcomes, and a third subgroup with subcortical/white matter involvement and variable outcomes. These findings suggest that ANPRA comprises multiple pathophysiologic or immunologic processes, and recognizing this may guide development of subgroup-specific therapies and more tailored prognostic counseling.

Forty-three percent of seronegative AE cases had persistent seizures at final follow-up, consistent with prior reports linking seronegativity to higher postencephalitis epilepsy risk.^16^ This suggests that patients with seronegative AE, particularly those with ANPRA, may require prolonged antiseizure medication.

Early immunotherapy was the most important modifiable prognostic factor. This was in line with the findings of Lee et al.,^11^ who found that adding rituximab and/or tocilizumab improved eventual outcomes. Similarly, in our analysis, patients receiving second-line immunotherapy achieved comparable median mRS outcomes to those managed with first-line agents alone despite higher baseline disability. Our findings highlight the importance of early treatment followed by appropriate escalation of immunotherapy.

The reported prevalence of seronegative AE varies across the literature, with estimates ranging from 7% to 55%, yet the number of published cases remains relatively limited.^11,17^ The lack of specific biomarkers could contribute to underdiagnosis and misdiagnosis. Although ANPRA and definite LE criteria are highly specific (>95%), a significant proportion of cases (15.8%) remain unclassified despite a strong suspicion of neuroinflammatory disease.^7^ Conversely, the lower specificity of possible AE criteria allows inclusion of mimics at this diagnostic workup stage in up to 46% of cases.^7^ Our experience of data extraction from published reports supported inconsistent application of seronegative AE diagnostic criteria and highlights that ‘possible’ AE is an entry-level criterion, not a firm diagnosis.

Cell-based assays used in routine diagnostic testing identify only known antigenic targets and cannot identify novel autoantibodies or alternative mechanisms, such as T cell–mediated immunopathogenesis. Screening serum and CSF from patients with seronegative AE using agnostic tests, such as live hippocampal neurons or brain slices, could increase diagnostic certainty.^18^ However, the interpretation of IHC and neuronal binding assays remains heterogeneous because there is currently no consensus on whether binding in the absence of a known antigenic target aligns with the established diagnostic criteria.

Our study has several limitations. Case reports and small series have the potential to introduce publication and selection bias with over-representation of atypical or severe cases. In addition, >50% of the patients in our cohort were from a single center,^11^ potentially limiting generalizability of our findings. Treatment data were inconsistently reported across studies, hindering detailed analyses of disease course and response. Outcome measures were likewise heterogeneous, predominantly reporting mRS scores and with inconsistent follow-up intervals, which limited our ability to perform time course analyses. Furthermore, some seronegative patients may be reclassified as antibody-positive with discovery of additional novel autoantigens. Finally, antibody results depend on assay performance. For example, in the context of paraneoplastic antigens, up to one-third of patients testing negative on commercial panels tested positive in research laboratories.^19^ This limitation extends to neuronal surface antibody testing, where up to 20% of commercial indirect immunofluorescence assays were subsequently identified as false negatives after further testing in a research laboratory.^20^ The precise antibody detection will, therefore, affect which patients are defined as seronegative after autoantibody testing. Similarly, although standard testing will exclude many alternative diagnoses, the precise pattern of testing differed between cases.

Larger, multicenter prospective studies are needed, with standardized, longitudinal reporting of outcomes, treatment protocols, and paraclinical findings, including the results of extended antibody panels, live-cell assays, CSF cytokines, and advanced MRI protocols. Parallel biomarker discovery efforts are essential to delineate biologically driven subgroups and design targeted therapies. Lessons from dementia research illustrate this well: clinical phenotypes often overlap, and only the introduction of biomarkers has enabled accurate stratification, refined prognostication, and mechanism-based treatment trials.^21^ Clinical trials targeting specific seronegative AE profiles and pathomechanisms will be critical to improve outcomes in this heterogeneous and often treatment-refractory condition. However, given the limited characterization of seronegative AE in the literature, this will require international collaborative studies. Nonetheless, some AE subtypes will likely remain too infrequent to permit fully individualized treatment approaches.

Seronegative AE is a heterogeneous condition with diverse clinical features and outcomes. Our findings highlight key phenotypic and outcome-associated subtypes and the importance of prompt, effective immunotherapy. Continued efforts toward biomarker discovery and longitudinal phenotyping are essential to improving diagnosis and stratifying treatment in this diagnostically challenging population.

## Glossary

AE: autoimmune encephalitis
ANPRA: antibody-negative probable AE
CASE: Clinical Assessment Scale for Autoimmune Encephalitis
LE: limbic encephalitis
mRS: modified Rankin Scale

## References

[1] Dalmau J, Graus F. Antibody-mediated encephalitis. N Engl J Med. 2018;378(9):840-851. doi:10.1056/NEJMra1708712 29490181

[2] van Steenhoven RW, Titulaer MJ. Seronegative autoimmune encephalitis: exploring the unknown. Brain. 2022;145(10):3339-3340. doi:10.1093/brain/awac338 36111366 PMC9586533

[3] Graus F, Titulaer MJ, Balu R, et al. A clinical approach to diagnosis of autoimmune encephalitis. Lancet Neurol. 2016;15(4):391-404. doi:10.1016/S1474-4422(15)00401-9 26906964 PMC5066574

[4] Dalmau J, Graus F. Diagnostic criteria for autoimmune encephalitis: utility and pitfalls for antibody-negative disease. Lancet Neurol. 2023;22(6):529-540. doi:10.1016/S1474-4422(23)00083-2 37210100

[5] Abboud H, Probasco JC, Irani S, et al. Autoimmune encephalitis: proposed best practice recommendations for diagnosis and acute management. J Neurol Neurosurg Psychiatry. 2021;92(7):757-768. doi:10.1136/jnnp-2020-325300 33649022 PMC8223680

[6] Dubey D, Pittock SJ, Kelly CR, et al. Autoimmune encephalitis epidemiology and a comparison to infectious encephalitis. Ann Neurol. 2018;83(1):166-177. doi:10.1002/ana.25131 29293273 PMC6011827

[7] Van Steenhoven RW, de Vries JM, Bruijstens AL, et al. Mimics of autoimmune encephalitis: validation of the 2016 clinical autoimmune encephalitis criteria. Neurol Neuroimmunol Neuroinflamm. 2023;10(6):e200148. doi:10.1212/NXI.0000000000200148 37582614 PMC10427145

[8] Irani SR, Bera K, Waters P, et al. N-methyl-D-aspartate antibody encephalitis: temporal progression of clinical and paraclinical observations in a predominantly non-paraneoplastic disorder of both sexes. Brain. 2010;133(Pt 6):1655-1667. doi:10.1093/brain/awq113 20511282 PMC2877907

[9] Elrefaey A, Mohamedelkhair A, Fahmy L, et al. The clinical, diagnostic and treatment spectrum of seropositive and seronegative autoimmune encephalitis: single-center cohort study of 51 cases and review of the literature. Clin Exp Neuroimmunol. 2024;15(4):186-200. doi:10.1111/cen3.12802

[10] Berger B, Hauck S, Runge K, Tebartz van Elst L, Rauer S, Endres D. Therapy response in seronegative versus seropositive autoimmune encephalitis. Front Immunol. 2023;14:1196110. doi:10.3389/fimmu.2023.1196110 37325671 PMC10264660

[11] Lee WJ, Lee HS, Kim DY, et al. Seronegative autoimmune encephalitis: clinical characteristics and factors associated with outcomes. Brain. 2022;145(10):3509-3521. doi:10.1093/brain/awac166 35512357

[12] Irani SR, Alexander S, Waters P, et al. Antibodies to Kv1 potassium channel-complex proteins leucine-rich, glioma inactivated 1 protein and contactin-associated protein-2 in limbic encephalitis, Morvan's syndrome and acquired neuromyotonia. Brain. 2010;133(9):2734-2748. doi:10.1093/brain/awq213 20663977 PMC2929337

[13] Binks SNM, Veldsman M, Handel AE, et al. Fatigue predicts quality of life after leucine‐rich glioma‐inactivated 1‐antibody encephalitis. Ann Clin Transl Neurol. 2024;11(4):1053-1058. doi:10.1002/acn3.52006 38303486 PMC11021603

[14] Lim JA, Lee ST, Moon J, et al. Development of the clinical assessment scale in autoimmune encephalitis. Ann Neurol. 2019;85(3):352-358. doi:10.1002/ana.25421 30675918

[15] Ceronie B, Strippel C, Uy C, et al. Immunotherapy-resistant neuropathic pain and fatigue predict quality-of-life in contactin-associated protein-like 2 antibody disease. Ann Neurol. 2025;97(3):521-528. doi:10.1002/ana.27177 39825737 PMC11831874

[16] Jia L, Kim CY, Pleshkevich M, et al. Long-term seizure outcomes in autoimmune encephalitis. Neurohospitalist. 2025;15(4):343-354. doi:10.1177/19418744251331650 PMC11962926 40182605

[17] Graus F, Escudero D, Oleaga L, et al. Syndrome and outcome of antibody-negative limbic encephalitis. Eur J Neurol. 2018;25(8):1011-1016. doi:10.1111/ene.13661 29667271 PMC6037545

[18] Ricken G, Schwaiger C, De Simoni D, et al. Detection methods for autoantibodies in suspected autoimmune encephalitis. Front Neurol. 2018;9:841. doi:10.3389/fneur.2018.00841 30364136 PMC6191500

[19] Déchelotte B, Muñiz-Castrillo S, Joubert B, et al. Diagnostic yield of commercial immunodots to diagnose paraneoplastic neurologic syndromes. Neurol Neuroimmunol Neuroinflamm. 2020;7(3):e701. doi:10.1212/nxi.0000000000000701 32170044 PMC7136063

[20] Ruiz-García R, Muñoz-Sánchez G, Naranjo L, et al. Limitations of a commercial assay as diagnostic test of autoimmune encephalitis. Front Immunol. 2021;12:691536. doi:10.3389/fimmu.2021.691536 34267758 PMC8276168

[21] Therriault J, Schindler SE, Salvadó G, et al. Biomarker-based staging of Alzheimer disease: rationale and clinical applications. Nat Rev Neurol. 2024;20(4):232-244. doi:10.1038/s41582-024-00942-2 38429551

